# Supplementary material for: Lipid classes in adipose tissues and liver differ between Shetland ponies and Warmblood horses
Source: PLoS One. 2019 Mar 21;14(3):e0207568. doi: 10.1371/journal.pone.0207568 (PMC6428305; doi:10.1371/journal.pone.0207568)
Supplement: S1 Table — (DOCX) [file pone.0207568.s001.docx]

**S1 Table. Descriptive data of equines**

To characterise experimental animals in more detail and set pre-selection criteria, we used body condition scoring (BSC) system according to Carroll et al. [1] and Cresty neck scoring (CNS) system according to Carter et al. [2]. Further, all animals were assessed for plasma adrenocorticotropic hormone (ACTH) to rule out pituitary pars intermedia dysfunction (PPID). Insulin dysregulation was excluded for all animals, as fasting serum insulin values were under the threshold of < 20 µU/ml after the combined glucose-insulin test (CGIT) performed according to Eiler et al. [3].

|  | Shetland ponies | Warmblood horses |
| --- | --- | --- |
| Sex | 6 geldings | 6 geldings |
| Age, years | 6 ± 3* | 10 ± 3* |
| Body weight, kg | 118 ± 29* | 589 ± 58* |
| Body condition score (BCS)^1^,  1-6 [1] | 3.7 (2.2/4.4)** | 3.6 (3.1/4.2)** |
| Cresty neck score (CNS)^1^,  0-5 [2] | 2.5 (0.8/3)** | 2 (1.5/2.3)** |
| Plasma adrenocorticotropic hormone (ACTH), pg/mL  Threshold: 50 pg/mL | 15.9 (12.8/17.1)** | 16.4 (15.1/19.6)** |
| Fasting serum insulin, µU/mL  Threshold: 20 µU/mL [3] | 4.19 (2.96/5.3)** | 5.78 (5.14/8.02)** |

***** Mean ± SD. ** Median (25^th^/75^th^ percentile). ^1^Mean median of two independent

evaluators.

1. Carroll CL, Huntington PJ. Body condition scoring and weight estimation of horses. Equine Vet J. 1988;20: 41-45.
2. Carter RA, Geor RJ, Staniar WB, Cubitt TA, Harris PA. Apparent adiposity assessed by standardised scoring systems and morphometric measurements in horses and ponies. Vet J. 2009;179: 204-210.
3. Eiler H, Frank N, Andrews FM, Oliver JW, Fecteau KA. Physiologic assessment of blood glucose homeostasis via combined intravenous glucose and insulin testing in horses. Am J Vet Res. 2005;66: 1598-1604.
